# *N*′-(4-Hy­droxy­benzyl­idene)-4-nitro­benzohydrazide

**DOI:** 10.1107/S1600536810042364

**Published:** 2010-10-23

**Authors:** Chun-Hua Dai, Fu-Lin Mao

**Affiliations:** a Jiangsu Provincial Key Laboratory of Coastal Wetland Bioresources and Environmental Protection, Department of Chemistry, Yancheng Teachers University, Yancheng 224002, People’s Republic of China

## Abstract

The title compound, C_14_H_11_N_3_O_4_, was prepared by the reaction of 4-nitro­benzohydrazide with 4-hy­droxy­benz­alde­hyde. The whole mol­ecule of the compound is approximately planar, with a mean deviation from the least-squares plane through all the non-H atoms of 0.050 (2) Å; the dihedral angle between the two benzene rings is 2.0 (2)°. In the crystal, the benzohydrazide mol­ecules are linked through inter­molecular O—H⋯O and N—H⋯O hydrogen bonds, forming layers in the *bc* plane.

## Related literature

For medical applications of hydrazones, see: Ajani *et al.* (2010 ▶); Zhang *et al.* (2010 ▶); Angelusiu *et al.* (2010 ▶). For related structures, see: Huang & Wu (2010 ▶); Khaledi *et al.* (2010 ▶); Zhou & Yang (2010 ▶); Ji & Lu (2010 ▶); Singh & Singh (2010 ▶); Ahmad *et al.* (2010 ▶).
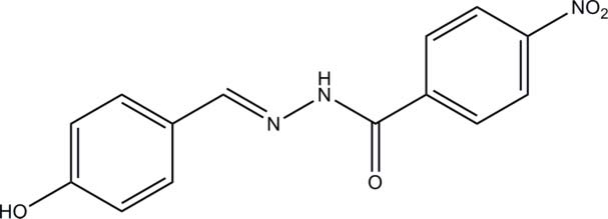


## Experimental

### 

#### Crystal data

C_14_H_11_N_3_O_4_
*M* *_r_* = 285.26
Monoclinic, 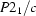

*a* = 7.856 (3) Å
*b* = 13.368 (5) Å
*c* = 12.527 (5) Å
β = 95.748 (4)°
*V* = 1309.0 (9) Å^3^
*Z* = 4
Mo *K*α radiation
μ = 0.11 mm^−1^
*T* = 298 K
0.18 × 0.17 × 0.17 mm

#### Data collection

Bruker SMART CCD area-detector diffractometer
Absorption correction: multi-scan (*SADABS*; Bruker, 2001 ▶) *T*_min_ = 0.981, *T*_max_ = 0.982
7505 measured reflections
2822 independent reflections
1736 reflections with *I* > 2σ(*I*)
*R*_int_ = 0.025

#### Refinement

*R*[*F* ^2^ > 2σ(*F* ^2^)] = 0.045
*wR*(*F* ^2^) = 0.119
*S* = 1.04
2822 reflections
194 parameters
1 restraint
H atoms treated by a mixture of independent and constrained refinement
Δρ_max_ = 0.16 e Å^−3^
Δρ_min_ = −0.16 e Å^−3^

### 

Data collection: *SMART* (Bruker, 2007 ▶); cell refinement: *SAINT* (Bruker, 2007 ▶); data reduction: *SAINT*; program(s) used to solve structure: *SHELXTL* (Sheldrick, 2008 ▶); program(s) used to refine structure: *SHELXTL*; molecular graphics: *SHELXTL*; software used to prepare material for publication: *SHELXTL*.

## Supplementary Material

Crystal structure: contains datablocks global, I. DOI: 10.1107/S1600536810042364/sj5045sup1.cif

Structure factors: contains datablocks I. DOI: 10.1107/S1600536810042364/sj5045Isup2.hkl

Additional supplementary materials:  crystallographic information; 3D view; checkCIF report

## Figures and Tables

**Table 1:** Hydrogen-bond geometry (Å, °)

*D*—H⋯*A*	*D*—H	H⋯*A*	*D*⋯*A*	*D*—H⋯*A*
O1—H1⋯O2^i^	0.82	1.98	2.7841 (18)	166
N2—H2⋯O4^ii^	0.90 (1)	2.24 (1)	3.094 (2)	159 (2)

Symmetry codes: (i) 
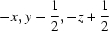
; (ii) 
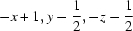
.

**Fig. 1.**
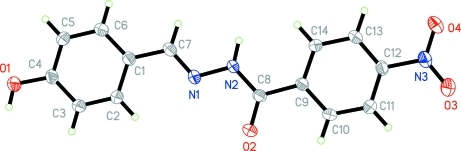
The molecular structure of the title compound showing 30% probability displacement ellipsoids and the atomic numbering.

**Fig. 2.**
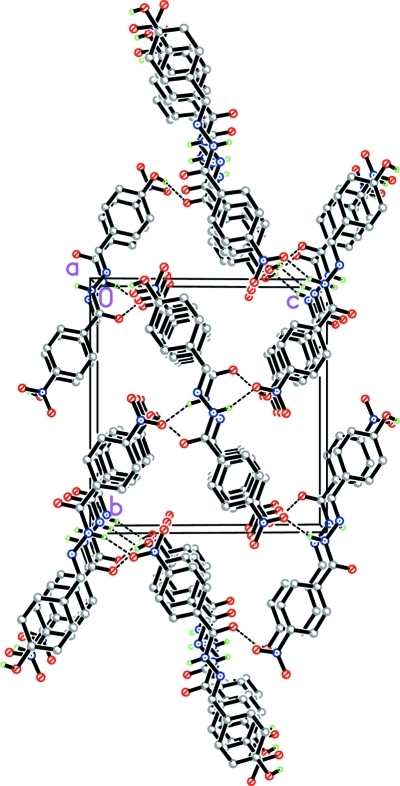
Crystal packing of the title compound, viewed down the a axis. Intermolecular interactions are drawn as dashed lines.

## supplementary crystallographic information

### Comment

Recently, medical applications of a number of hydrazone compounds have been reported  (Ajani *et al.*, 2010; Zhang *et al.*, 2010; Angelusiu *et al.*, 2010). Structural  investigations of several hydrazone derivatives have also been determined (Huang & Wu, 2010; Khaledi *et al.*, 2010; Zhou & Yang, 2010; Ji & Lu, 2010; Singh & Singh, 2010; Ahmad *et al.*, 2010). In this paper, we report the structure of the new derivative  *N*'-(4-Hydroxybenzylidene)-4-nitrobenzohydrazide (I).

The whole molecule of (I) is approximately planar, with a mean deviation from the least squares plane through all 21 non-hydrogen atoms of 0.050 (2) Å; the dihedral angle between the C1···C6 and C9···C14 benzene rings is 2.0 (2)°. The bond lengths and angles are comparable to those found in the hydrazone compounds cited above. In the crystal structure, the hydrazone molecules are linked through intermolecular O1–H1···O2 and N2–H2···O4 hydrogen bonds (Table 1), to form two-dimensional layers in the *bc* plane, as shown in Fig. 2.

### Experimental

The reaction of 4-nitrobenzohydrazide (0.181 g, 1 mmol) with 4-hydroxybenzaldehyde (0.122 g, 1 mmol) in 50 ml methanol at room temperature afforded the title compound. Single crystals were formed by slow evaporation of the clear solution in air.

### Refinement

The amino H atom was located in a difference Fourier map and refined with N–H = 0.90 (1) Å, and *U*_iso_ = 0.08 Å^2^. Other H atoms were positioned geometrically (C–H = 0.93 Å, O–H = 0.82 Å) and refined as riding with *U*_iso_(H) = 1.2*U*_eq_(C) or 1.5 *U*_eq_(O).

### Crystal data

**Table:**

C_14_H_11_N_3_O_4_	*F*(000) = 592
*M**_r_* = 285.26	*D*_x_ = 1.447 Mg m^−^^3^
Monoclinic, *P*2_1_/*c*	Mo *K*α radiation, λ = 0.71073 Å
Hall symbol: -P 2ybc	Cell parameters from 1632 reflections
*a* = 7.856 (3) Å	θ = 2.2–26.0°
*b* = 13.368 (5) Å	µ = 0.11 mm^−^^1^
*c* = 12.527 (5) Å	*T* = 298 K
β = 95.748 (4)°	Block, yellow
*V* = 1309.0 (9) Å^3^	0.18 × 0.17 × 0.17 mm
*Z* = 4	

### Data collection

**Table:**

Bruker SMART CCD area-detector diffractometer	2822 independent reflections
Radiation source: fine-focus sealed tube	1736 reflections with *I* > 2σ(*I*)
graphite	*R*_int_ = 0.025
ω scans	θ_max_ = 27.0°, θ_min_ = 2.2°
Absorption correction: multi-scan (*SADABS*; Bruker, 2001)	*h* = −9→10
*T*_min_ = 0.981, *T*_max_ = 0.982	*k* = −17→14
7505 measured reflections	*l* = −16→14

### Refinement

**Table:**

Refinement on *F*^2^	Primary atom site location: structure-invariant direct methods
Least-squares matrix: full	Secondary atom site location: difference Fourier map
*R*[*F*^2^ > 2σ(*F*^2^)] = 0.045	Hydrogen site location: inferred from neighbouring sites
*wR*(*F*^2^) = 0.119	H atoms treated by a mixture of independent and constrained refinement
*S* = 1.04	*w* = 1/[σ^2^(*F*_o_^2^) + (0.0494*P*)^2^ + 0.1504*P*] where *P* = (*F*_o_^2^ + 2*F*_c_^2^)/3
2822 reflections	(Δ/σ)_max_ < 0.001
194 parameters	Δρ_max_ = 0.16 e Å^−^^3^
1 restraint	Δρ_min_ = −0.16 e Å^−^^3^

### Special details

**Table:**

Geometry. All e.s.d.'s (except the e.s.d. in the dihedral angle between two l.s. planes) are estimated using the full covariance matrix. The cell e.s.d.'s are taken into account individually in the estimation of e.s.d.'s in distances, angles and torsion angles; correlations between e.s.d.'s in cell parameters are only used when they are defined by crystal symmetry. An approximate (isotropic) treatment of cell e.s.d.'s is used for estimating e.s.d.'s involving l.s. planes.
Refinement. Refinement of *F*^2^ against ALL reflections. The weighted *R*-factor *wR* and goodness of fit *S* are based on *F*^2^, conventional *R*-factors *R* are based on *F*, with *F* set to zero for negative *F*^2^. The threshold expression of *F*^2^ > σ(*F*^2^) is used only for calculating *R*-factors(gt) *etc*. and is not relevant to the choice of reflections for refinement. *R*-factors based on *F*^2^ are statistically about twice as large as those based on *F*, and *R*- factors based on ALL data will be even larger.

### Fractional atomic coordinates and isotropic or equivalent isotropic displacement parameters (Å^2^)

**Table:**

	*x*	*y*	*z*	*U*_iso_*/*U*_eq_	
N1	0.20526 (19)	−0.05033 (11)	0.04617 (11)	0.0433 (4)	
N2	0.2594 (2)	0.02540 (11)	−0.01727 (11)	0.0420 (4)	
N3	0.4845 (2)	0.44506 (12)	−0.22229 (13)	0.0510 (4)	
O1	0.0039 (2)	−0.46112 (9)	0.25453 (11)	0.0598 (4)	
H1	−0.0521	−0.4411	0.3021	0.090*	
O2	0.17995 (17)	0.13969 (9)	0.09997 (10)	0.0512 (4)	
O3	0.4755 (2)	0.52833 (11)	−0.18407 (13)	0.0872 (6)	
O4	0.54671 (18)	0.42836 (10)	−0.30610 (10)	0.0603 (4)	
C1	0.1654 (2)	−0.22332 (13)	0.07612 (14)	0.0395 (4)	
C2	0.1046 (2)	−0.20626 (13)	0.17594 (14)	0.0433 (5)	
H2A	0.1006	−0.1412	0.2020	0.052*	
C3	0.0511 (2)	−0.28366 (13)	0.23572 (14)	0.0444 (5)	
H3	0.0107	−0.2708	0.3017	0.053*	
C4	0.0567 (2)	−0.38146 (13)	0.19834 (14)	0.0424 (5)	
C5	0.1185 (2)	−0.39987 (13)	0.10044 (15)	0.0463 (5)	
H5	0.1240	−0.4651	0.0753	0.056*	
C6	0.1721 (2)	−0.32147 (13)	0.04010 (14)	0.0446 (5)	
H6	0.2132	−0.3346	−0.0256	0.053*	
C7	0.2197 (2)	−0.13939 (13)	0.01282 (14)	0.0422 (4)	
H7	0.2649	−0.1512	−0.0519	0.051*	
C8	0.2426 (2)	0.12013 (13)	0.01678 (13)	0.0372 (4)	
C9	0.3070 (2)	0.20201 (12)	−0.05002 (13)	0.0352 (4)	
C10	0.2986 (2)	0.29875 (13)	−0.01120 (14)	0.0454 (5)	
H10	0.2541	0.3099	0.0538	0.055*	
C11	0.3552 (2)	0.37861 (13)	−0.06733 (15)	0.0482 (5)	
H11	0.3492	0.4435	−0.0411	0.058*	
C12	0.4209 (2)	0.36016 (13)	−0.16304 (14)	0.0395 (4)	
C13	0.4312 (2)	0.26543 (14)	−0.20412 (14)	0.0449 (5)	
H13	0.4760	0.2548	−0.2691	0.054*	
C14	0.3738 (2)	0.18647 (13)	−0.14713 (14)	0.0446 (5)	
H14	0.3799	0.1219	−0.1740	0.054*	
H2	0.299 (3)	0.0083 (16)	−0.0797 (11)	0.080*	

### Atomic displacement parameters (Å^2^)

**Table:**

	*U*^11^	*U*^22^	*U*^33^	*U*^12^	*U*^13^	*U*^23^
N1	0.0554 (10)	0.0363 (9)	0.0402 (9)	−0.0019 (7)	0.0149 (7)	0.0061 (7)
N2	0.0593 (10)	0.0337 (8)	0.0360 (8)	−0.0025 (7)	0.0187 (7)	0.0034 (7)
N3	0.0596 (11)	0.0466 (10)	0.0482 (10)	−0.0082 (8)	0.0127 (8)	0.0080 (8)
O1	0.0868 (11)	0.0365 (7)	0.0615 (9)	−0.0022 (7)	0.0338 (8)	0.0061 (7)
O2	0.0688 (9)	0.0451 (8)	0.0442 (8)	0.0005 (7)	0.0273 (6)	−0.0002 (6)
O3	0.1470 (17)	0.0418 (9)	0.0802 (12)	−0.0201 (10)	0.0472 (11)	0.0006 (8)
O4	0.0700 (10)	0.0636 (9)	0.0511 (9)	−0.0088 (7)	0.0247 (7)	0.0110 (7)
C1	0.0415 (10)	0.0392 (10)	0.0385 (10)	0.0004 (8)	0.0075 (8)	0.0030 (8)
C2	0.0554 (11)	0.0332 (10)	0.0429 (10)	−0.0023 (8)	0.0127 (9)	−0.0005 (8)
C3	0.0553 (11)	0.0411 (10)	0.0390 (10)	0.0011 (9)	0.0154 (9)	−0.0003 (9)
C4	0.0467 (11)	0.0362 (10)	0.0453 (11)	0.0007 (8)	0.0100 (9)	0.0077 (9)
C5	0.0585 (12)	0.0326 (9)	0.0495 (11)	0.0012 (9)	0.0135 (9)	−0.0022 (9)
C6	0.0524 (11)	0.0432 (11)	0.0400 (10)	0.0015 (9)	0.0142 (9)	−0.0008 (9)
C7	0.0472 (11)	0.0441 (11)	0.0367 (10)	−0.0006 (9)	0.0123 (8)	0.0042 (9)
C8	0.0394 (10)	0.0405 (10)	0.0327 (9)	0.0025 (8)	0.0084 (8)	0.0015 (8)
C9	0.0367 (9)	0.0369 (9)	0.0330 (9)	0.0032 (7)	0.0083 (7)	0.0026 (8)
C10	0.0589 (12)	0.0430 (11)	0.0373 (10)	−0.0008 (9)	0.0197 (9)	−0.0045 (8)
C11	0.0632 (12)	0.0353 (10)	0.0481 (11)	−0.0029 (9)	0.0159 (10)	−0.0030 (9)
C12	0.0424 (10)	0.0371 (10)	0.0400 (10)	−0.0021 (8)	0.0088 (8)	0.0068 (8)
C13	0.0554 (11)	0.0457 (11)	0.0363 (10)	0.0012 (9)	0.0180 (9)	0.0024 (9)
C14	0.0605 (12)	0.0343 (9)	0.0417 (10)	0.0032 (9)	0.0186 (9)	−0.0013 (8)

### Geometric parameters (Å, °)

**Table:**

N1—C7	1.271 (2)	C4—C5	1.386 (2)
N1—N2	1.3803 (19)	C5—C6	1.382 (2)
N2—C8	1.347 (2)	C5—H5	0.9300
N2—H2	0.899 (9)	C6—H6	0.9300
N3—O3	1.217 (2)	C7—H7	0.9300
N3—O4	1.2220 (19)	C8—C9	1.496 (2)
N3—C12	1.471 (2)	C9—C10	1.386 (2)
O1—C4	1.364 (2)	C9—C14	1.388 (2)
O1—H1	0.8200	C10—C11	1.376 (2)
O2—C8	1.2247 (19)	C10—H10	0.9300
C1—C6	1.390 (2)	C11—C12	1.374 (2)
C1—C2	1.401 (2)	C11—H11	0.9300
C1—C7	1.462 (2)	C12—C13	1.372 (2)
C2—C3	1.368 (2)	C13—C14	1.375 (2)
C2—H2A	0.9300	C13—H13	0.9300
C3—C4	1.391 (2)	C14—H14	0.9300
C3—H3	0.9300		
			
C7—N1—N2	117.08 (14)	C1—C6—H6	119.5
C8—N2—N1	117.50 (13)	N1—C7—C1	120.02 (15)
C8—N2—H2	124.6 (15)	N1—C7—H7	120.0
N1—N2—H2	117.9 (15)	C1—C7—H7	120.0
O3—N3—O4	123.30 (16)	O2—C8—N2	122.05 (15)
O3—N3—C12	118.13 (16)	O2—C8—C9	120.45 (15)
O4—N3—C12	118.55 (16)	N2—C8—C9	117.49 (14)
C4—O1—H1	109.5	C10—C9—C14	118.85 (15)
C6—C1—C2	118.08 (16)	C10—C9—C8	117.14 (14)
C6—C1—C7	121.71 (15)	C14—C9—C8	124.01 (15)
C2—C1—C7	120.20 (16)	C11—C10—C9	121.04 (16)
C3—C2—C1	121.08 (16)	C11—C10—H10	119.5
C3—C2—H2A	119.5	C9—C10—H10	119.5
C1—C2—H2A	119.5	C12—C11—C10	118.37 (17)
C2—C3—C4	120.32 (16)	C12—C11—H11	120.8
C2—C3—H3	119.8	C10—C11—H11	120.8
C4—C3—H3	119.8	C13—C12—C11	122.28 (16)
O1—C4—C5	118.02 (16)	C13—C12—N3	119.15 (15)
O1—C4—C3	122.60 (15)	C11—C12—N3	118.56 (16)
C5—C4—C3	119.38 (16)	C12—C13—C14	118.62 (15)
C6—C5—C4	120.15 (17)	C12—C13—H13	120.7
C6—C5—H5	119.9	C14—C13—H13	120.7
C4—C5—H5	119.9	C13—C14—C9	120.83 (16)
C5—C6—C1	120.97 (16)	C13—C14—H14	119.6
C5—C6—H6	119.5	C9—C14—H14	119.6

### Hydrogen-bond geometry (Å, °)

**Table:**

*D*—H···*A*	*D*—H	H···*A*	*D*···*A*	*D*—H···*A*
O1—H1···O2^i^	0.82	1.98	2.7841 (18)	166
N2—H2···O4^ii^	0.90 (1)	2.24 (1)	3.094 (2)	159 (2)

Symmetry codes: (i) −*x*, *y*−1/2, −*z*+1/2; (ii) −*x*+1, *y*−1/2, −*z*−1/2.
